# Broad range molecular detection methods identify only *Borrelia* spp. in erythema migrans biopsies and blood of tick-bitten patients

**DOI:** 10.1016/j.onehlt.2024.100886

**Published:** 2024-08-30

**Authors:** Philippe Pérot, Laura Tondeur, Sara Moutailler, Delphine Chrétien, Nicole Corre-Catelin, Muriel Vayssier-Taussat, Marc Eloit, Catherine Chirouze, Céline Cazorla, OHTICKS Consortium, Laurence Arowas, Elisabeth Botelho-Nevers, Céline Cazorla, Catherine Chirouze, Delphine Chrétien, Nicole Corre-Catelin, Marc Eloit, Pascale Frey-Klett, Arnaud Fontanet, Clémence Galon, Karine Lacombe, Sara Moutailler, Philippe Pérot, Véronique Perronne, Valentine Piquard, Marie Préau, Costanza Puppo, Laura Tondeur, Marie-Noelle Ungeheuer, Muriel Vayssier-Taussat, Ayla Zayoud

**Affiliations:** a Pathogen Discovery Laboratory, Institut Pasteur, Université Paris Cité, F-75015 Paris, France; b Institut Pasteur, Université Paris Cité, The WOAH (OIE) Collaborating Center for the Detection and Identification in Humans of Emerging Animal Pathogens, F-75015 Paris, France; c Emerging Infectious Diseases Unit, Institut Pasteur, Paris, France; d ANSES, INRAE, Ecole Nationale Vétérinaire d'Alfort, UMR BIPAR, Laboratoire de Santé Animale, Maisons-Alfort, F-94700, France; e Institut Pasteur, Université Paris Cité, Clinical Investigation and Access to Research Bioresources (ICAReB) Platform, Paris, France; f Animal Health Division, INRAE, 37380 Nouzilly, France; g Ecole Nationale Vétérinaire d'Alfort, F-94700 Maisons-Alfort, France; h Chrono-Environnement UMR6249, CNRS, Université Bourgogne Franche-Comté, F-25000 Besançon, France; i Infectious Disease Department, University Hospital of Besançon, F-25000, France; j Infectious Disease department, University Hospital of Saint Etienne, Avenue Albert-Raimond, 42055, Saint Etienne Cedex 02, France

**Keywords:** Ticks, Lyme disease, Erythema migrans, Metagenomics, RT-PCR, Pathogen discovery

## Abstract

In this multicenter study conducted in France, we challenged the hypothesis of the transmission of pathogens other than *Borrelia* spp. in 22 patients developing erythema migrans following a tick bite. Using a combination of high-throughput microfluidic PCRs and agnostic metagenomics on skin biopsies and blood samples, no microorganisms other than *Borrelia* spp. was found.

## 1 Introduction

Lyme borreliosis (LB) is the most common tick-borne disease in Europe, is caused by different genospecies of *Borrelia burgdorferi* sensu lato (Bbsl) complex [1,2] and its early clinical diagnosis is based on the presence of cutaneous erythema migrans (EM) [3]. *Borrelia burgdorferi* sensu lato bacteria, the causative agents of Lyme borreliosis, are transmitted in Europe mainly by the tick species *Ixodes ricinus.* Other tick species could be involved in maintaining the transmission cycle of this bacteria in wildlife but are rarely involved in the transmission to humans [4,5]. *Ixodes ricinus* can also transmit other infectious agents pathogenic for humans: bacteria such as *Anaplasma phagocytophilum*, virus such as Tick-borne Encephalitis, or parasites such as *Babesia* [[6], [7], [8], [9], [10]]. In western Europe, the estimated annual incidence of LB is 22/100000 inhabitants with a wide variation depending on the country [11]. However, incomplete surveillance, missed diagnoses and the use of insensitive laboratory tests suggest significant underreporting. In 2022, approximately 63,000 cases of LD were reported to the CDC in the USA. Recent estimates using different methods suggest that around 476,000 people could be diagnosed with Lyme disease each year in the United States [12].

A minority of patients adequately treated by antibiotics against Lyme borreliosis complains about polymorphic, non-specific (asthenia, fever, myalgia) but persistent symptoms [13]. One hypothesis to explain this clinic persistency is an infection by unforeseen or novel pathogenic microorganisms transmitted by the tick bite. In this context, we proposed a multi-disciplinary project, OHTICKS, bringing together veterinarians, physicians, scientists and sociologists, to better characterize the tick-borne diseases present in humans and domestic animals following a tick bite, in a One Health approach. Four French university hospitals were involved in the OHTICKS project (Besançon, Saint-Etienne, Saint-Antoine (AP-HP) and Garches (AP-HP)). They were chosen either because they were located in areas with a high incidence of Lyme disease, or because they had particular expertise in this field.

The main objective of OHTICKS consortium was to identify neglected, unsuspected or new micro-organisms in patients bitten by ticks, using two complementary approaches: (i) high-throughput microfluidic real-time PCR targeting 41 major tick-borne bacteria and parasites known to circulate in Europe; (ii) random deep sequencing (NGS) expanding the search to unexpected or novel tick-borne pathogens (bacteria, parasites and viruses). In this study, we used EM, which is specific enough for a clinical diagnosis of Lyme disease, as a marker of transmission of *Borrelia* spp. or other pathogens. In these patients, small in number but well characterized, we searched for *Borrelia* spp. and other infectious agents in one cutaneous biopsy, plus plasma and blood pellet sampled at Day 0 (concomitant with the biopsy) and Day 14, using these two methods.

## 2 Materials and methods

Twenty-six patients with suspected EM were included in the OHTICKS protocol. The diagnosis was made by infectiologists of the consortium, and the photos of all EM were blindly reviewed by an infectiologist from outside the inclusion centers. The lesions were classified as: “suggestive of EM”, defined as skin lesion greater than 5 cm and centrifugal evolution with peripheral border darker than the center of the lesion (*N* = 12/26, 46 %); “not very typical”, defined as skin lesion greater than 5 cm and homogeneous erythema (*N* = 11/26, 42 %); and “really not typical”, defined as skin lesion less than 5 cm and homogeneous erythema (*N* = 3/26, 11 %, excluded from the analysis). The “really not typical” EM patients were initially enrolled in the study because they had reported a tick bite and had a little redness at the site of the bite. In conditions of arthropod bite, differential diagnoses were: hypersensitivity reactions at the site of the bite (non-infectious) or infections with pyogenes bacteria following bites; outside the context of the bite: fixed pigmented erythema, dermatophytosis, granuloma annulare and Morpheus patch. One skin biopsy was eventually performed from the active EM area of each of the 22 patients (*N* = 12/22 “suggestive”, *N* = 10/22 “not very typical”). Among these 22 subjects, 13 (59 %) subjects were male and the median [IQR] age was 49 [40–64] years. Thirteen (59 %) patients had removed a tick from the site of EM (*N* = 6/13 “suggestive”; *N* = 7/13 “not very typical”). Sixteen (73 %) lesions were located on the lower limbs, 3 (14 %) on the upper limbs and 3 (14 %) on the trunk (Table 1). Demographic characteristics did not differ between the 2 groups, except for the variable “bite in the garden” (*p* = 0.03). Clinically, only lesion size was almost significantly greater in the “suggestive” group (median [IQR] = 13 [9–19]cm) than in the “not very typical” group (median [IQR] = 8 [6–15]cm) (*p* = 0.06) (Table 1), as expected by the classification criteria. The geographical distribution of tick bites of the 22 patients is given in Table 2.

**Table 1:** Clinical and biological characteristics of the 22 patients.

	“Suggestive EM†” group *N* = 12	“Not typical EM†” group *N* = 10	Total *N* = 22	pvalue
**Gender,***n (%)*				
*Male*	9 (75.0)	4 (40.0)	13 (59.1)	0.19
*Female*	3 (25.0)	6 (60.0)	9 (40.9)	
**Age (years),***median (Q1-Q3)*	52 [42–70]	47 [36–60]	49 [40–64]	0.58
**Presence of a tick bite,***n (%)*	10 (83.3)	8 (80.0)	18 (81.8)	0.99
**Place of suspected tick bite,***n (%)*				
*Forest*	9 (75.0)	5 (50.0)	14 (63.6)	0.38
*Meadow*	2 (16.7)	3 (30.0)	5 (22.7)	0.62
*Garden*	0 (0)	4 (40.0)	4 (18.2)	0.03
*Other*	2 (16.7)	1 (10.0)	3 (13.6)	0.99
**Appearance of ticks when bitten*,****n (%)*				
*Large and white (swallowed)*	0 (0)	2 (20.0)	2 (9.1)	0.50
*Small and black (not swallowed)*	6 (50.0)	5 (50.0)	11 (50.0)	
*Missing data*	6 (50.0)	3 (30.0)	9 (40.9)	
**Time to onset of ME after tick bite*,****n (%)*				
*Less than a week*	2 (16.7)	2 (20.0)	4 (18.2)	0.91
*Between 1 and 2 weeks*	3 (25.0)	1 (10.0)	4 (18.2)	
*More than 2 weeks*	6 (50.0)	6 (60.0)	12 (54.6)	
*Missing data*	1 (8.3)	1 (10.0)	2 (9.1)	
**Anatomical location of ME*,****n (%)*				
*Upper limb*	1 (8.3)	2 (20.0)	3 (13.6)	0.39
*Lower limb*	8 (66.7)	8 (80.0)	16 (72.7)	
*Trunk*	3 (25.0)	0 (0)	3 (13.6)	
**Size of lesion (cms)**	13 [9–19]	8 [6–15]	10 [7–15]	0.06
**Clinical signs at inclusion,***n (%)*				
*Asthenia*	2 (16.7)	3 (30.0)	5 (22.7)	0.62
*Neurological signs*	2 (16.7)	4 (40.0)	6 (27.3)	0.35
*Rheumatological signs*	2 (16.7)	2 (20.0)	4 (18.2)	0.99
*Sensory disorders*	0 (0)	1 (10.0)	1 (4.6)	0.46
*Other clinical signs*	1 (8.3)	4 (40.0)	5 (22.7)	0.14
**Prescription of antibiotic treatment following inclusion visit,***n (%)*	12 (100.0)	10 (100.0)	22 (100.0)	
**Detection of Borreliella,***n (%)*	2 (16.7)	3 (30.0)	5 (22.7)	0.62

† EM: Erythema Migrans.

**Table 2:** Geographical distribution of tick bites of the 22 patients.

French department		French region	“Suggestive EM†” group N = 12 *n (%)*	“Not typical EM†” group N = 10 *n (%)*	Total N = 22 *n (%)*
25	Doubs	Bourgogne-Franche-Comté	1 (8.3)	1 (10.0)	2 (9.0)
28	Eure-et-Loir	Centre-Val de Loire	1 (8.3)	0 (0)	1 (4.6)
39	Jura	Bourgogne-Franche-Comté	0 (0)	2 (20.0)	2 (9.0)
42	Loire	Auvergne- Rhone-Alpes	7 (58.4)	4 (40.0)	11 (50.0)
70	Haute-Saône	Bourgogne-Franche-Comté	1 (8.3)	0 (0)	1 (4.6)
72	Sarthe	Pays de la Loire	0 (0)	1 (10.0)	1 (4.6)
74	Haute-Savoie	Auvergne- Rhone-Alpes	1 (8.3)	0 (0)	1 (4.6)
77	Seine et Marne	Ile de France	0 (0)	1 (10.0)	1 (4.6)
Missing data			1 (8.3)	1 (10.0)	2 (9.0)

† EM: Erythema Migrans*.*

### 2.1 Real-time microfluidic PCR

To search for the presence of 41 known tick-borne pathogens (bacteria and parasites among the genera *Borrelia*, *Anaplasma*, *Rickettsia*, *Ehrlichia*, *Bartonella*, *Coxiella*, *Francisella*, *Babesia*, *Theileria*), the 22 skin biopsies (DNA and RNA), 26 plasma samples (D0 and D14) and 26 blood pellet (D0 and D14) from patients were screened using a high-throughput real-time microfluidic PCRs [14]. Briefly, a 48.48 dynamic array were screened using the BioMark™ real-time PCR system (Standard Biotools, CA, USA). This chip can be used to perform 2304 real-time microfluidic PCR reactions thanks to 48 PCR mixes and 48 samples placed into individual wells, prior to transfer into individual chambers for the reaction. The thermal cycling conditions were 50 °C for 2 mins, 95 °C for 10 mins, and 40 cycles at 95 °C for 15 s and 60 °C for 1 min. One negative water control, one inhibitory molecule control (*Escherichia coli* EDL933 strain), and one DNA extraction control (human targeting primers) were added to each chip. Then positive results were confirmed by Nested PCR.

### 2.2 NGS

To complete the first analysis and look for the presence of common or unexpected pathogens transmitted by tick bite, we carried out a pathogen discovery approach based on random deep sequencing, as described previously in a context of routine analyses of patient's samples in a clinical setting [15]. Briefly, total RNA from skin biopsies taken from the active area of 22 patients presenting with erythema migrans were sequenced using the SMARTer Stranded Total RNA-Seq Kit v2 Pico Input Mammalian. RNA sequencing allows for identification of all types of microorganisms, as all express their genomes as RNA transcripts. Plasma samples from 26 patients (including the 22 patients who had skin biopsy) collected at the time of their first medical visit (D0, concomitant with skin biopsies) and two weeks later (D14) were treated by nucleases before total nucleic acids extraction to enrich for nucleic acids associated with bacterial bodies or virus particles, then sequenced using an adapted MALBAC protocol [16]. In parallel, total RNA extracted from blood pellets at D0 and D14 were sequenced using the SMARTer Stranded Total RNA-Seq Kit v2. Sequencing was done on Illumina NextSeq500 and NovaSeq instruments with average depth of 58 million clusters per sample. The search for microbial sequences was done using Kraken2 [17] on NCBI nt (version 2021-3-29) and a complementary search for viruses was achieved through Microseek [18].

## 3 Results

We first screened the 22 skin biopsies (RNA and DNA extracts), plus plasma (total nucleic acids) and blood pellets (RNA) collected at the time of the first medical visit (D0, concomitant with skin biopsies) and two weeks later (D14), for the presence of 41 tick-borne pathogens (bacteria and parasites) using a high-throughput real-time microfluidic PCR. On the 22 skin biopsies RNA extracts, one was positive and confirmed as *Borrelia afzelii*. Regarding the DNA analysis, although 14 samples were positive for *Borrelia* spp. using the microfluidic PCR, only 5 could be confirmed as *Borrelia afzelii* through Sanger sequencing (*N* = 2/5 “suggestive of EM”; *N* = 3/5 “not very typical”), including the sample that was positive following the RNA approach. All skin biopsies were negative for the other tick-borne pathogens tested. None of the 41 tick-borne pathogens were detected from plasma and blood pellets.

We then carried out an agnostic pathogen identification approach based on random deep sequencing. On the 22 skin biopsies sequenced (total RNA only), five (22 %) were positive for *Borrelia burgdorferi* sensu lato, matching the 5 samples that were positive by the real-time microfluidic PCR from DNA, with 4 out of 5 being attributed to the species *Borrelia afzelii* (the last one attributed to the genus *Borrelia*). Other skin biopsies were negative for *Borrelia* spp., and no other viral, bacterial or fungal sequences were found. Plasma samples at D0 and D14 were all positive for Torque Teno Virus (TTV, *Anelloviridae*), a non-pathogenic virus whose load is proposed as a marker of immune status. TTV viral loads were therefore quantified using the TTV R-GENE real-time PCR assay (BioMérieux SA, Marcy l'Etoile, France), showing an average of 2.4 log_10_ Cp/mL and a distribution of viral loads not associated with immune dysfunction or increased risk of infection [19,20], with no difference between D0 and D14. A few other viral reads were detected by NGS in certain plasma samples: STL Polyomavirus (*N* = 14/22, 63 %), Merkel Cell Polyomavirus (*N* = 7/22, 31 %) and Epstein-Barr Virus (6/22, 27 %) but confirmatory specific PCR were all negative, suggesting that STLPyV, MCPyV corresponded to skin flora and that EBV viremia was better detected by NGS than by PCR. No microbial sequences were detected from blood pellets.

## 4 Discussion

We challenged the hypothesis of the transmission of unexpected or novel pathogen other than *Borrelia* spp. in tick-bitten patients developing erythema migrans, using a combination of high-throughput real-time microfluidic PCRs and agnostic deep sequencing. No evidence of microorganisms other than *Borrelia* spp. was found in this small but well characterized cohort. The significance of these results is nevertheless limited by the fact that *Borrelia* spp. was identified only in a minority of samples, leaving open the possibility that other etiologies may also have escaped our investigation.

## Ethics statement

This work was approved on December 18, 2017 by the Comité de Protection des Personnes (CPP) Est-II under protocol number CPP 17/567 (ANSM reference 2017-A02916–47). The CPP in France has a role similar to that of Ethics Committees or Institutional Review Boards (IRBs) in other countries. Volunteers received an information sheet detailing the study, and informed consent was obtained before their participation.

## Funding

The OHTICKS study was funded by the French Agence Nationale de la Recherche (ANR) grant number ANR-16-CES35–0011.

## CRediT authorship contribution statement

**Philippe Pérot:** Writing – review & editing, Writing – original draft, Validation, Methodology, Investigation. **Laura Tondeur:** Writing – review & editing, Writing – original draft, Resources, Methodology, Data curation. **Sara Moutailler:** Writing – review & editing, Writing – original draft, Validation, Methodology, Investigation, Conceptualization. **Delphine Chrétien:** Validation, Methodology, Investigation. **Nicole Corre-Catelin:** Resources, Project administration, Data curation. **Muriel Vayssier-Taussat:** Writing – review & editing, Supervision, Project administration, Methodology, Investigation, Funding acquisition, Conceptualization. **Marc Eloit:** Writing – review & editing, Writing – original draft, Supervision, Methodology, Investigation, Funding acquisition, Conceptualization. **Catherine Chirouze:** Writing – review & editing, Resources, Methodology, Investigation, Conceptualization. **Céline Cazorla:** Writing – review & editing, Writing – original draft, Resources, Methodology, Investigation, Conceptualization. **Céline Cazorla:** Writing – review & editing, Writing – original draft, Resources, Methodology, Investigation, Conceptualization.

## Declaration of competing interest

The authors declare they have no conflicts of interest.

## Data Availability

Data will be made available on request.
